# Supplementary material for: An open-source framework for large-scale, flexible evaluation of biomedical text mining systems
Source: J Biomed Discov Collab. 2008 Jan 29;3:1. doi: 10.1186/1747-5333-3-1 (PMC2276192; doi:10.1186/1747-5333-3-1)
Supplement: Additional file 1 — Evaluation platform source code. The additional file contains the software evaluation framework discussed in this paper. The most current release can be downloaded from SourceForge.net [1]. See the accompanying README file for instructions on installation and use. Also included in the distribution is a collection of UIMA wrappers for some commonly used BioNLP tools and annotated corpora. [file 1747-5333-3-1-S1.zip › bionlp_uima_repository-1.0.1/docs/api/overview-tree.html]

Class Hierarchy (Center for Computational Pharmacology Util/UIMA API)


|  |  |  |
| --- | --- | --- |
| **Overview** \| Package \| Class \| **Tree** \| **Deprecated** \| **Index** \| **Help** | |  |
| PREV   NEXT | **FRAMES**    **NO FRAMES**     **All Classes** |  |


---


## Hierarchy For All Packages

**Package Hierarchies:**: edu.uchsc.ccp.uima.ae.nlp.anaphora, edu.uchsc.ccp.uima.ae.nlp.ei.gene, edu.uchsc.ccp.uima.ae.nlp.ei.mutation, edu.uchsc.ccp.uima.ae.nlp.opendmap, edu.uchsc.ccp.uima.ae.nlp.parser, edu.uchsc.ccp.uima.ae.nlp.sentenceDetector, edu.uchsc.ccp.uima.ae.nlp.stemmer, edu.uchsc.ccp.uima.ae.nlp.tokenizer, edu.uchsc.ccp.uima.ae.util, edu.uchsc.ccp.uima.ae.util.converter, edu.uchsc.ccp.uima.ae.util.filter, edu.uchsc.ccp.uima.annotation, edu.uchsc.ccp.uima.annotation.syntactic, edu.uchsc.ccp.uima.cc.summary, edu.uchsc.ccp.uima.cc.summary.comparison, edu.uchsc.ccp.uima.cc.summary.opendmap, edu.uchsc.ccp.uima.mention, edu.uchsc.ccp.uima.util, edu.uchsc.ccp.uima.util.opendmap, edu.uchsc.ccp.util, edu.uchsc.ccp.util.external.abgene, edu.uchsc.ccp.util.external.abner, edu.uchsc.ccp.util.external.geniatagger, edu.uchsc.ccp.util.external.kex, edu.uchsc.ccp.util.external.lingpipe, edu.uchsc.ccp.util.external.opennlp, edu.uchsc.ccp.util.external.penn, edu.uchsc.ccp.util.external.stanfordparser, edu.uchsc.ccp.util.nlp, edu.uchsc.ccp.util.nlp.annotation, edu.uchsc.ccp.util.nlp.annotation.comparison, edu.uchsc.ccp.util.nlp.annotation.filter, edu.uchsc.ccp.util.nlp.annotation.printer, edu.uchsc.ccp.util.nlp.document, edu.uchsc.ccp.util.nlp.ei, edu.uchsc.ccp.util.nlp.ei.xml, edu.uchsc.ccp.util.nlp.mention, edu.uchsc.ccp.util.nlp.mention.comparison, edu.uchsc.ccp.util.nlp.parser.corpus, edu.uchsc.ccp.util.nlp.stats, edu.uchsc.ccp.util.nlp.stemmer, edu.uchsc.ccp.util.nlp.tokenizer, edu.uchsc.ccp.util.xml

---

## Class Hierarchy

- java.lang.**Object**
  - edu.uchsc.ccp.util.external.abgene.**AbGeneEISystem** (implements edu.uchsc.ccp.util.nlp.ei.EIFileBasedSystem)- edu.uchsc.ccp.util.external.abner.**Abner\_Util**- edu.uchsc.ccp.util.nlp.annotation.**Annotation\_Util**- edu.uchsc.ccp.util.nlp.annotation.comparison.**AnnotationComparator** (implements java.util.Comparator<T>)
          - edu.uchsc.ccp.util.nlp.annotation.comparison.**EntityPartsAnnotationComparator**
          - edu.uchsc.ccp.uima.cc.summary.comparison.**AnnotationGroup**- edu.uchsc.ccp.uima.cc.summary.comparison.**AnnotationGroupID** (implements java.lang.Comparable<T>)- edu.uchsc.ccp.uima.util.opendmap.**AnnotationMap**- edu.uchsc.ccp.util.nlp.annotation.**AnnotationSet** (implements java.lang.Comparable<T>)- edu.uchsc.ccp.uima.util.opendmap.**AnnotationString**- edu.uchsc.ccp.util.nlp.annotation.printer.**AnnotationToFileOutput** (implements edu.uchsc.ccp.util.nlp.annotation.printer.AnnotationPrinterUtil)- edu.uchsc.ccp.util.nlp.annotation.**Annotator** (implements java.lang.Comparable<T>)- org.apache.uima.analysis\_engine.annotator.Annotator\_ImplBase (implements org.apache.uima.analysis\_engine.annotator.BaseAnnotator)
                          - org.apache.uima.analysis\_engine.annotator.JTextAnnotator\_ImplBase (implements org.apache.uima.analysis\_engine.annotator.JTextAnnotator)
                            - edu.uchsc.ccp.uima.ae.nlp.ei.gene.**AbGene\_AE**- edu.uchsc.ccp.uima.ae.nlp.ei.gene.**ABNER\_AE**- edu.uchsc.ccp.uima.ae.util.filter.**AnnotationConsensusFilter\_AE**- edu.uchsc.ccp.uima.ae.util.**AnnotationFromFileLoader\_AE**- edu.uchsc.ccp.uima.ae.util.filter.**AnnotationSetFilter\_AE**- edu.uchsc.ccp.uima.ae.util.converter.**AnnotationSetOverrider\_AE**- edu.uchsc.ccp.uima.ae.util.converter.**AnnotatorConverter\_AE**- edu.uchsc.ccp.uima.ae.util.filter.**AnnotatorFilter\_AE**- edu.uchsc.ccp.uima.ae.util.converter.**AnnotatorOverrider\_AE**- edu.uchsc.ccp.uima.ae.util.converter.**ClassMentionConverter\_AE**- edu.uchsc.ccp.uima.ae.util.filter.**ClassMentionRemovalFilter\_AE**- edu.uchsc.ccp.uima.ae.util.filter.**ClassMentionTypeFilter\_AE**- edu.uchsc.ccp.uima.ae.util.converter.**DocumentLevelAnnotationCreator\_AE**- edu.uchsc.ccp.uima.ae.nlp.tokenizer.**GeniaTaggerOutputFileLoader\_AE**- edu.uchsc.ccp.uima.ae.nlp.ei.gene.**KeX\_AE**- edu.uchsc.ccp.uima.ae.nlp.sentenceDetector.**KeXSentenceDetector\_AE**- edu.uchsc.ccp.uima.ae.nlp.ei.gene.**LingPipeEntityTagger\_AE**- edu.uchsc.ccp.uima.ae.nlp.tokenizer.**LingPipePOSTagger\_AE**- edu.uchsc.ccp.uima.ae.nlp.sentenceDetector.**LingPipeSentenceDetector\_AE**- edu.uchsc.ccp.uima.ae.nlp.ei.mutation.**MutationFinder\_AE**- edu.uchsc.ccp.uima.ae.nlp.opendmap.**OpenDmapAnnotator**
                                                                      - edu.uchsc.ccp.uima.ae.nlp.opendmap.**CCPOpenDMAP\_AE**
                                                                      - edu.uchsc.ccp.uima.ae.nlp.sentenceDetector.**OpenNLPSentenceDetector\_AE**- edu.uchsc.ccp.uima.ae.util.filter.**OverlappingAnnotationResolver\_AE**- edu.uchsc.ccp.uima.ae.nlp.ei.gene.**PennBioTagger\_AE**- edu.uchsc.ccp.uima.ae.nlp.tokenizer.**PennBioTokenizer\_AE**- edu.uchsc.ccp.uima.ae.nlp.stemmer.**PorterStemmer\_AE**- edu.uchsc.ccp.uima.ae.nlp.anaphora.**ProteinAnaphoraResolver**- edu.uchsc.ccp.uima.ae.util.filter.**SlotRemovalFilter\_AE**- edu.uchsc.ccp.uima.ae.nlp.parser.**StanfordParser\_AE**
                          - edu.uchsc.ccp.uima.cc.summary.comparison.**ComparatorConfigurator**- edu.uchsc.ccp.uima.cc.summary.comparison.**ComparisonGroup**- edu.uchsc.ccp.util.nlp.parser.corpus.**CorpusParser** (implements java.util.Iterator<E>)
                                - edu.uchsc.ccp.util.nlp.parser.corpus.**Bio1CorpusParser**- edu.uchsc.ccp.util.nlp.parser.corpus.**BioIECorpusParser**- edu.uchsc.ccp.util.nlp.parser.corpus.**GeniaCorpusParser**- edu.uchsc.ccp.util.nlp.parser.corpus.**IProLinkCorpus1Parser**- edu.uchsc.ccp.util.nlp.parser.corpus.**IProLinkCorpus2Parser**- edu.uchsc.ccp.util.nlp.parser.corpus.**TexasCorpusParser**- edu.uchsc.ccp.util.nlp.parser.corpus.**YapexCorpusParser**
                                - edu.uchsc.ccp.util.nlp.annotation.printer.**DefaultPrintOutput** (implements edu.uchsc.ccp.util.nlp.annotation.printer.AnnotationPrinterUtil)- edu.uchsc.ccp.uima.util.opendmap.**DMAPStanfordPhraseGraph** (implements edu.uchsc.ccp.opendmap.DMAPPropertyFunctionHandler)- edu.uchsc.ccp.opendmap.DMAPTokenizer (implements java.lang.Iterable<T>, java.util.Iterator<E>)
                                      - edu.uchsc.ccp.uima.util.opendmap.**DMAPJcasTokenizer**
                                      - edu.uchsc.ccp.util.nlp.annotation.comparison.**DocumentLevelAnnotationCreator**- edu.uchsc.ccp.util.nlp.document.**DocumentSection**- edu.uchsc.ccp.util.nlp.document.**DocumentUtil**- org.apache.uima.cas.impl.FeatureStructureImpl (implements java.lang.Cloneable, org.apache.uima.cas.FeatureStructure)
                                              - org.apache.uima.jcas.cas.TOP
                                                - org.apache.uima.jcas.cas.AnnotationBase (implements org.apache.uima.cas.AnnotationBaseFS)
                                                  - org.apache.uima.jcas.tcas.Annotation (implements org.apache.uima.cas.text.AnnotationFS)
                                                    - edu.uchsc.ccp.uima.annotation.**CCPAnnotation**
                                                      - edu.uchsc.ccp.uima.annotation.**CCPDocumentAnnotation**
                                                        - edu.uchsc.ccp.uima.annotation.**CCPDocumentInformation**- edu.uchsc.ccp.uima.annotation.**CCPDocumentSection**- edu.uchsc.ccp.uima.annotation.**CCPDocumentSubSection**
                                                        - edu.uchsc.ccp.uima.annotation.**CCPTextAnnotation**
                                                          - edu.uchsc.ccp.uima.annotation.**CCPSemanticAnnotation**- edu.uchsc.ccp.uima.annotation.**CCPSyntacticAnnotation**
                                                              - edu.uchsc.ccp.uima.annotation.syntactic.**CCPClauseAnnotation**- edu.uchsc.ccp.uima.annotation.syntactic.**CCPPhraseAnnotation**- edu.uchsc.ccp.uima.annotation.syntactic.**CCPSentenceAnnotation**- edu.uchsc.ccp.uima.annotation.syntactic.**CCPTokenAnnotation**
                                                  - edu.uchsc.ccp.uima.annotation.**CCPAnnotationSet**- edu.uchsc.ccp.uima.annotation.**CCPAnnotator**- edu.uchsc.ccp.uima.mention.**CCPMention**
                                                        - edu.uchsc.ccp.uima.mention.**CCPClassMention**- edu.uchsc.ccp.uima.mention.**CCPSlotMention**
                                                            - edu.uchsc.ccp.uima.mention.**CCPComplexSlotMention**- edu.uchsc.ccp.uima.mention.**CCPNonComplexSlotMention**
                                                        - edu.uchsc.ccp.uima.annotation.**CCPSpan**- edu.uchsc.ccp.uima.annotation.syntactic.**CCPTypedDependency**
                                              - edu.uchsc.ccp.util.nlp.document.**GenericDocument**- edu.uchsc.ccp.util.nlp.**GenericTextUtils**- edu.uchsc.ccp.util.external.geniatagger.**GeniaTaggerOutputFileParser**- edu.uchsc.ccp.util.nlp.parser.corpus.**IProLinkParser**- edu.uchsc.ccp.util.external.kex.**KeXEISystem** (implements edu.uchsc.ccp.util.nlp.ei.EIFileBasedSystem)- edu.uchsc.ccp.util.nlp.**KeyValuePair** (implements java.lang.Comparable<T>)- edu.uchsc.ccp.util.external.lingpipe.**LingPipe\_Util**- edu.uchsc.ccp.util.nlp.mention.**Mention** (implements java.lang.Comparable<T>)
                                                              - edu.uchsc.ccp.util.nlp.mention.**ClassMention**- edu.uchsc.ccp.util.nlp.mention.**ComplexSlotMention**- edu.uchsc.ccp.util.nlp.mention.**SlotMention**
                                                              - edu.uchsc.ccp.util.nlp.mention.comparison.**MentionComparator** (implements java.util.Comparator<T>)
                                                                - edu.uchsc.ccp.util.nlp.mention.comparison.**IdenticalMentionComparator**
                                                                  - edu.uchsc.ccp.util.nlp.mention.comparison.**IdenticalClassMentionComparator** (implements edu.uchsc.ccp.util.nlp.mention.comparison.ClassMentionComparator)- edu.uchsc.ccp.util.nlp.mention.comparison.**IdenticalComplexSlotMentionComparator** (implements edu.uchsc.ccp.util.nlp.mention.comparison.ComplexSlotMentionComparator)- edu.uchsc.ccp.util.nlp.mention.comparison.**IdenticalSlotMentionComparator** (implements edu.uchsc.ccp.util.nlp.mention.comparison.SlotMentionComparator)
                                                                - edu.uchsc.ccp.util.nlp.mention.**MentionDocumentLevelSortComparator** (implements java.util.Comparator<T>)- edu.uchsc.ccp.util.nlp.mention.**MentionSortComparator** (implements java.util.Comparator<T>)- edu.uchsc.ccp.util.nlp.stats.**NLPStats**- edu.uchsc.ccp.util.external.opennlp.**OpenNLPSentenceSegmenter**- edu.uchsc.ccp.util.nlp.annotation.filter.**OverlappingAnnotationResolver**- edu.uchsc.ccp.util.external.penn.**PennBioTaggerEISystem** (implements edu.uchsc.ccp.util.nlp.ei.EISystem)- edu.uchsc.ccp.util.external.penn.**PennBioTokenizer\_Util** (implements edu.uchsc.ccp.util.nlp.tokenizer.Tokenizer)- org.tartarus.PorterStemmer
                                                                                - edu.uchsc.ccp.util.nlp.stemmer.**PorterStemmer\_Util**
                                                                                - edu.uchsc.ccp.util.nlp.stats.**PRFResult**- edu.uchsc.ccp.uima.util.opendmap.**ReferenceProcessor**- org.apache.uima.resource.Resource\_ImplBase (implements org.apache.uima.resource.Resource)
                                                                                      - org.apache.uima.resource.ConfigurableResource\_ImplBase (implements org.apache.uima.resource.ConfigurableResource)
                                                                                        - org.apache.uima.collection.CasConsumer\_ImplBase (implements org.apache.uima.collection.CasConsumer)
                                                                                          - edu.uchsc.ccp.uima.cc.summary.comparison.**AnnotationComparator\_CC** (implements org.apache.uima.collection.base\_cpm.CasObjectProcessor)- edu.uchsc.ccp.uima.cc.summary.**AnnotationPrinter** (implements org.apache.uima.collection.base\_cpm.CasObjectProcessor)- edu.uchsc.ccp.uima.cc.summary.opendmap.**TestPrinter** (implements org.apache.uima.collection.base\_cpm.CasObjectProcessor)
                                                                                      - edu.uchsc.ccp.util.**RunExecutable**- edu.uchsc.ccp.util.nlp.annotation.printer.**SimpleCollectionReaderFormatOutput** (implements edu.uchsc.ccp.util.nlp.annotation.printer.AnnotationPrinterUtil)- edu.uchsc.ccp.util.nlp.annotation.**Span** (implements java.lang.Comparable<T>)- edu.uchsc.ccp.util.nlp.annotation.comparison.**SpanComparator** (implements java.util.Comparator<T>)
                                                                                              - edu.uchsc.ccp.util.nlp.annotation.comparison.**IgnoreSpanComparator**- edu.uchsc.ccp.util.nlp.annotation.comparison.**SharedEndSpanComparator**- edu.uchsc.ccp.util.nlp.annotation.comparison.**SharedStartOrEndSpanComparator**- edu.uchsc.ccp.util.nlp.annotation.comparison.**SharedStartSpanComparator**- edu.uchsc.ccp.util.nlp.annotation.comparison.**SloppySpanComparator**- edu.uchsc.ccp.util.nlp.annotation.comparison.**StrictSpanComparator**
                                                                                              - edu.uchsc.ccp.util.external.stanfordparser.**StanfordParser\_Util**- edu.uchsc.ccp.uima.util.opendmap.**TestSummarizer**- edu.uchsc.ccp.util.nlp.annotation.**TextAnnotation** (implements java.lang.Comparable<T>)- java.lang.**Throwable** (implements java.io.Serializable)
                                                                                                      - java.lang.**Exception**
                                                                                                        - edu.uchsc.ccp.util.nlp.annotation.**InvalidSpanException**- edu.uchsc.ccp.util.external.stanfordparser.**InvalidTypedDependencyException**
                                                                                                      - org.apache.uima.jcas.cas.TOP\_Type
                                                                                                        - org.apache.uima.jcas.cas.AnnotationBase\_Type
                                                                                                          - org.apache.uima.jcas.tcas.Annotation\_Type
                                                                                                            - edu.uchsc.ccp.uima.annotation.**CCPAnnotation\_Type**
                                                                                                              - edu.uchsc.ccp.uima.annotation.**CCPDocumentAnnotation\_Type**
                                                                                                                - edu.uchsc.ccp.uima.annotation.**CCPDocumentInformation\_Type**- edu.uchsc.ccp.uima.annotation.**CCPDocumentSection\_Type**- edu.uchsc.ccp.uima.annotation.**CCPDocumentSubSection\_Type**
                                                                                                                - edu.uchsc.ccp.uima.annotation.**CCPTextAnnotation\_Type**
                                                                                                                  - edu.uchsc.ccp.uima.annotation.**CCPSemanticAnnotation\_Type**- edu.uchsc.ccp.uima.annotation.**CCPSyntacticAnnotation\_Type**
                                                                                                                      - edu.uchsc.ccp.uima.annotation.syntactic.**CCPClauseAnnotation\_Type**- edu.uchsc.ccp.uima.annotation.syntactic.**CCPPhraseAnnotation\_Type**- edu.uchsc.ccp.uima.annotation.syntactic.**CCPSentenceAnnotation\_Type**- edu.uchsc.ccp.uima.annotation.syntactic.**CCPTokenAnnotation\_Type**
                                                                                                          - edu.uchsc.ccp.uima.annotation.**CCPAnnotationSet\_Type**- edu.uchsc.ccp.uima.annotation.**CCPAnnotator\_Type**- edu.uchsc.ccp.uima.mention.**CCPMention\_Type**
                                                                                                                - edu.uchsc.ccp.uima.mention.**CCPClassMention\_Type**- edu.uchsc.ccp.uima.mention.**CCPSlotMention\_Type**
                                                                                                                    - edu.uchsc.ccp.uima.mention.**CCPComplexSlotMention\_Type**- edu.uchsc.ccp.uima.mention.**CCPNonComplexSlotMention\_Type**
                                                                                                                - edu.uchsc.ccp.uima.annotation.**CCPSpan\_Type**- edu.uchsc.ccp.uima.annotation.syntactic.**CCPTypedDependency\_Type**
                                                                                                        - edu.uchsc.ccp.util.external.stanfordparser.**TypedDependencyCls**- edu.uchsc.ccp.util.external.stanfordparser.**TypedDependencyCls\_Util**- edu.uchsc.ccp.uima.util.**UIMA\_Annotation\_Util**- edu.uchsc.ccp.uima.util.**UIMA\_Util**- edu.uchsc.ccp.uima.util.**UIMASyntacticAnnotation\_Util**- edu.uchsc.ccp.util.nlp.ei.xml.**XMLEntityExtractor**- edu.uchsc.ccp.util.xml.**XMLValidityChecker**

## Interface Hierarchy

- edu.uchsc.ccp.util.nlp.annotation.printer.**AnnotationPrinterUtil**- edu.uchsc.ccp.util.nlp.mention.comparison.**ClassMentionComparator**- edu.uchsc.ccp.util.nlp.mention.**ClassMentionTypes**- edu.uchsc.ccp.util.nlp.mention.comparison.**ComplexSlotMentionComparator**- edu.uchsc.ccp.util.nlp.mention.**ComplexSlotMentionTypes**- edu.uchsc.ccp.util.nlp.document.**DocumentSectionTypes**- edu.uchsc.ccp.util.nlp.ei.**EISystem**
              - edu.uchsc.ccp.util.nlp.ei.**EIFileBasedSystem**
              - edu.uchsc.ccp.util.nlp.mention.comparison.**SlotMentionComparator**- edu.uchsc.ccp.util.nlp.mention.**SlotMentionTypes**- edu.uchsc.ccp.util.nlp.tokenizer.**Tokenizer**

---


|  |  |  |
| --- | --- | --- |
| **Overview** \| Package \| Class \| **Tree** \| **Deprecated** \| **Index** \| **Help** | |  |
| PREV   NEXT | **FRAMES**    **NO FRAMES**     **All Classes** |  |


---
